# Sensing of Catecholamine in Human Urine Using a Simple Colorimetric Assay Based on Direct Melanochrome and Indolequinone Formation

**DOI:** 10.3390/s23083971

**Published:** 2023-04-13

**Authors:** Mariagrazia Lettieri, Michele Spinelli, Laura Caponi, Simona Scarano, Pasquale Palladino, Angela Amoresano, Maria Minunni

**Affiliations:** 1 Department of Chemistry ‘Ugo Schiff’, University of Florence, 50019 Sesto Fiorentino, Italy; 2 Department of Chemical Sciences, University of Naples Federico II, 80126 Naples, Italy; 3 Laboratory of Clinical Pathology, University Hospital of Pisa, 56126 Pisa, Italy; 4 INBB—Istituto Nazionale Biostrutture e Biosistemi, Consorzio Interuniversitario, 00136 Rome, Italy

**Keywords:** Parkinson’s disease, therapeutic drug monitoring, urine analysis, levodopa, dopamine, norepinephrine, melanochrome, indolequinone, catecholamines oxidation, colorimetric assay

## Abstract

We used the first enzyme-free synthesis and stabilization of soluble melanochrome (MC) and 5,6-indolequinone (IQ) derived from levodopa (LD), dopamine (DA), and norepinephrine (NE) oxidation to develop a simple colorimetric assay for catecholamine detection in human urine, also elucidating the time-dependent formation and molecular weight of MC and IQ using UV–Vis spectroscopy and mass spectrometry. The quantitative detection of LD and DA was achieved in human urine using MC as a selective colorimetric reporter to demonstrate the potential assay applicability in a matrix of interest in therapeutic drug monitoring (TDM) and in clinical chemistry. The assay showed a linear dynamic range between 5.0 mg L^−1^ and 50.0 mg L^−1^, covering the concentration range of DA and LD found in urine samples from, e.g., Parkinson’s patients undergoing LD-based pharmacological therapy. The data reproducibility in the real matrix was very good within this concentration range (RSD_av_% 3.7% and 6.1% for DA and LD, respectively), also showing very good analytical performances with the limits of detection of 3.69 ± 0.17 mg L^−1^ and 2.51 ± 0.08 mg L^−1^ for DA and LD, respectively, thus paving the way for the effective and non-invasive monitoring of dopamine and levodopa in urine from patients during TDM in Parkinson’s disease.

## 1. Introduction

The development of a simple colorimetric assay is at the forefront of (bio)analysis as a result of its outstanding merits for point of care and spot-testing for real-time sensing with simplified procedures, and the reduced time and cost of the analyses [1,2,3].

Recently, we focused our work on the development of colorimetric (and fluorescence-based) methods for the quantification of analytes of different molecular weights, within matrices of increasing complexity, i.e., chlorine in water, sugars in beer wort, p-nitrophenol in urine, polyphenols and anthocyanins in foods, proteins in human serum and urine, and catecholamines in therapeutic drugs. In particular, the development of simple, rapid, and low-cost assays that work in real matrices as urine for drug monitoring in clinical chemistry is of relevance. We recently developed a colorimetric assay for the selective detection and quantification of levodopa in the co-presence of carbidopa, and vice versa, in some pharmaceutical formulations for the treatment of patients with Parkinson’s disease (PD) [4,5], i.e., an age-associated progressive neurodegenerative disorder affecting millions of people worldwide but still without a cure other than the pharmacological treatment of symptoms using the synthetic catecholamine levodopa (LD) to restore the concentration of the naturally occurring neurotransmitter dopamine (DA) in the brain in order to improve the quality of life of patients [6,7,8].

The method was based on the discovery of the capability of magnesium cation in dimethyl sulfoxide to trigger the development of a purple/blue color in the presence of levodopa or dopamine, whereas this color was absent, in the same environment, for norepinephrine (NE) and for synthetic catecholamine analogues carbidopa (CD) and benserazide [5]. We ascribed the purple/blue color to melanochrome (MC) formation, an elusive intermediate stage of catecholamine polymerization, previously generated through enzymatic oxidation of LD, 5,6-dihydroxyindole (DHI) or 5,6-dihydroxyindole-2-carboxylic acid (DHICA) [9,10,11,12,13,14] and described as biindolyls isomers generated from the nucleophilic attack of DHI molecule on the electrophile molecule 5,6-indolequinone (IQ) [12,13,14].

In this work, we develop the study of the MC and IQ formation to better define their use as colorimetric reporters in the quantitative detection of catecholamines. Then, we move on to the application of the assay to human urine samples to demonstrate its potential applicability in LD therapeutic drug monitoring (TDM). The uncertainty regarding the precise structure of MC arose from its high instability when obtained with previous methods that required the chemical reduction and the acetylation of the colored precipitate to achieve soluble fractions for the analyses, impairing the direct investigation of the molecule [12,13,14]. Analogously, any previous attempt to isolate and characterize the IQ failed, and indirect chemical evidence of an indolequinone formation was obtained through the oxidation of analogue model molecules with protected reactive positions that conferred a longer stability to the transient intermediates [15,16,17,18,19]. Here, instead, we took the advantage of the first enzyme-free synthesis and stabilization in Mg^2+^/DMSO at basic pH of both soluble MC (λ_max_ 540–600 nm), derived from the oxidation of DA or its synthetic analogue LD, and soluble IQ (λ_max_ 340–360 nm) derived from the oxidation of norepinephrine (NE). Accordingly, it was possible to shed light on their molecular weight (MW) and time evolution through the direct examination of such compounds by means of LC-MS/MS-based analysis and using UV–Vis spectroscopy, confirming the MW indicated for the enzymatic product for the purple/blue melanochrome [12]. The MC was the main reaction product, with a m/z value 293.1 [M+H]^+^, for synthesis starting from DA or LD, although with slightly different kinetics, as highlighted by visible spectroscopy, therefore, indicating the decarboxylation of the latter monomer during the synthesis. Differently, according to an m/z value 147.2 (M+H)^+^ and the UV–Vis spectra, we found that the reaction product of NE, in the same condition, was mainly the IQ, without melanochrome formation, underlining the main influence of the β-hydroxyl group on the oxidative pathway of NE. The oxidative pathway of catecholamines, leading to melanochrome and indolequinone formation, is appreciably time-dependent, due to several steps of oxidation, intra-, and intermolecular reactions [15,16,17,18,19]. Such kinetics were here analyzed using visible spectroscopy, and by recording the absorbance values over time at 340 nm, which is associated with IQ formation, and 590 nm, which is associated with MC.

We believe that stable melanochrome generation and quantification in situ could facilitate the quantification of DA and LD for monitoring the LD-based therapy in PD. To this end, we evaluated the MC formation for the first time in human urine samples fortified with DA and LD with concentrations within the range found in urine samples from Parkinson’s patients undergoing LD-based pharmacological therapy, obtaining very good analytical performances (RSD_av_% 3.7% and LOD 3.69 ± 0.17 mg L^−1^ for DA, RSD_av_% 6.1% and LOD 2.51 ± 0.08 mg L^−1^ for LD). Accordingly, such selective synthesis of melanochrome in a real matrix appears to be very useful for the effective and non-invasive monitoring of dopamine and levodopa in urine, helping to develop more effective pharmacological treatments of the symptoms, avoiding inappropriate dosages or toxic effects, that could become part of the Internet of Things in healthcare, as discussed recently [20].

## 2. Materials and Methods

### 2.1. The Chemicals

Magnesium acetate tetrahydrate, ammonium chloride, hydrochloric acid, dimethyl sulfoxide, dopamine hydrochloride, norepinephrine, levodopa, and all mass spectrometry solvents and reagents were obtained from Sigma-Aldrich (Milan, Italy). Artificial urine (AU) was obtained from LCTech GmbH (Obertaufkirchen, Germany). AU composition: pH 6.6 ± 0.1, 25.00 g L^−1^ urea, 9.00 g L^−1^ sodium chloride, 2.50 g L^−1^ potassium dihydrogen orthophosphate, 2.50 g L^−1^ disodium hydrogen orthophosphate anhydrous, 3.00 g L^−1^ sodium sulphite hydrated, 3.00 g L^−1^ ammonium chloride and 2.00 g L^−1^ creatinine.

### 2.2. Standard Solutions and Urine Samples

Standard mixtures of levodopa, dopamine, and norepinephrine for quantitative analysis were prepared via the serial dilution of each catecholamine in a proper buffer (150 mM Mg(Ac)_2_, 150 mM NH_4_Cl at pH 9.4 in DMSO:H_2_O 1:1 (*v*/*v*)) [4]. The same buffer was used to dilute 1:8 (*v*/*v*) artificial urine or human urine samples from volunteers that did not consume levodopa-based drugs. The urine samples were spiked with a known amount of catecholamine spanning from 5.0 to 50.0 mg L^−1^ to simulate post-drug administered urine specimens [21,22].

### 2.3. Visible Spectroscopy

Absorbance studies were performed at 25 °C using Ocean View VIS-NIR (Maybachstrasse, Germany). The samples were analyzed in cuvettes with an optical path length of 1.0 cm. Time-dependent studies were performed on levodopa, dopamine, and norepinephrine standard mixtures, recording the visible spectra up to 144 min. Absorbance measurements of spiked urine samples were performed in 96-well microplate using Thermo Scientific Multiskan GO Microplate Spectrophotometer (Fisher scientific, Rodano (MI)) for the assessment of matrix effect in a quantitative bioanalytical assay. All colorimetric data from catecholamines oxidation within urine samples were fitted using the linear equation

A_585nm_ = m × C + a (1)

where A_585nm_ represents the absorbance at 585 nm due to formation of a purple/blue melanochrome, m represents the slope of the calibration curve, C represents the catecholamine concentration, and a represents the absorbance of unspiked urine samples at 585 nm (blank). The limit of detection (LOD) and the limit of quantification (LOQ) were calculated based on the standard deviation (SD) of the mean of the blank values (unspiked urine samples), as 3 × SD/m and 10 × SD/m, respectively, where m indicates the slope of the calibration curve. The assay reproducibility is reported as (mean) relative standard deviation % (RSD_av_%).

### 2.4. LC–MS/MS

The mass analysis was performed by injecting 1 µL of standard mixtures of dopamine, levodopa, and norepinephrine via HPLC auto sampler of a 6420 triple quadrupole system with an HPLC 1100 series binary pump from Agilent Technologies equipped with a C18 reverse phase column from Supelco. The samples were eluted (starting 1 min after injection) with a linear gradient of eluent B (0.1% formic acid and 5 mM ammonium formate in methanol) in A (0.1% formic acid and 5 mM ammonium formate in water) from 10% to 80% in 4 min. The column was re-equilibrated at the initial conditions for 4 min. A mass spectrometry method analysis based on multiple reaction monitoring (MRM) tandem mass spectrometry was set up to analyze the samples using a turbo ion spray source operating in positive-ion mode.

## 3. Results and Discussion

We first report the results achieved on the direct MC and IQ formation from catecholamines oxidation, respectively, dopamine (DA), levodopa (LD) forming MC and NE, IQ, instead. The LC-MS/MS and UV–Vis spectroscopic studies identified these products and showed the kinetics of their formation in solution after catecholamines addition. These findings allow the further use of MC and IQ as colorimetric reporters in the catecholamines quantitative detection. Furthermore, the direct MC formation is here demonstrated for the first time in human urine samples, paving the way to quantitative detection of DA and LD in this matrix. To that end, DA and LD spiked urine samples have been analyzed using the colorimetric assay based on MC formation and detection at 585 nm.

### 3.1. LC–MS/MS Analysis

Dopamine standard mixture at 50 µg L^−1^ was used for the optimization of the MRM transitions of melanochrome (MC), while an analogue norepinephrine solution was used for the optimization of IQ analysis. The samples were automatically tuned, accurately selecting ionization polarity, production, and collision energy (CE). A MS/Full Scan analysis was performed to find precursor ions of the target molecules. Subsequently, the selection of tandem mass spectral parameters improved the precursor ion detection. Finally, fragmentation experiments were performed at different collision energy (CE) values for identification of the products of reaction (see Table 1). Figure S1 (see Supplementary Materials) reports the MRM transitions of MC derived from DA and LD, where the coelution of the selected precursor/fragment ion pairs, using the most intense one for quantification, and the others as qualifiers of the products can be observed. The chromatograms indicates that MC eluted before DA or LD. NE generated IQ molecules only, whereas MC was not detected.

### 3.2. Spectroscopic Characterization of Products

**Figure 1:**
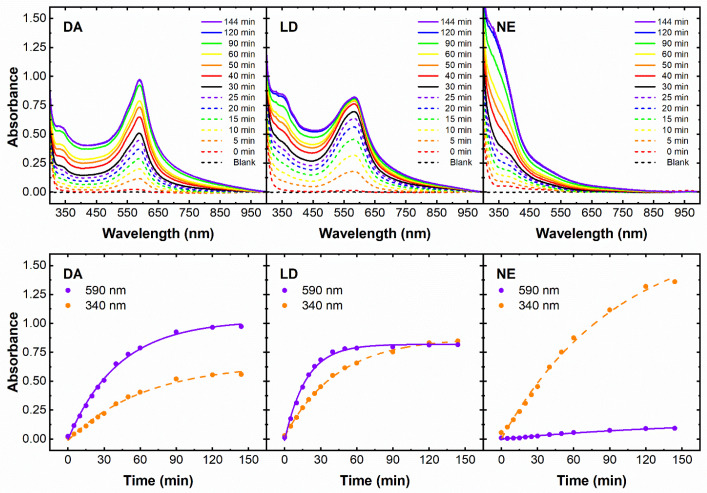
Time-dependent oxidation of natural catecholamines 0.2 g L^−1^ in DMSO:H_2_O 1:1 (*v*/*v*) in the presence of 150 mM Mg(Ac)_2_, 150 mM NH4Cl at pH 9.4 and 25 °C between 2 min and 144 min. UV–Vis spectra (upper panels) and absorbance values (lower panels) at 340 nm (orange circles/dashed line) and 590 nm (purple circles/dashed lines) for dopamine (DA), levodopa (LD), and norepinephrine (NE).

The UV–Vis spectra in Figure 1 indicate that the products of the oxidation of dopamine or levodopa in Mg^2+^/DMSO at basic pH are strictly related, although not identical in shape and relative intensity of the bands, and very different from the products of the oxidation of norepinephrine in the same conditions.

Dopamine products gave a broad absorbance with a main peak in the visible region corresponding to the λ_max_ value found for the blue 2,2′-DHI-dimer (590–600 nm) [10], plus a barely visible shoulder at the λ_max_ value, which was previously associated with the purple 2,4′- and 2,7′-DHI-dimer (540 nm) as well as with the melanochrome formed through levodopa oxidation [9,10,11,12,13,14]. Spectra from LD were similar to those from DA but the two visible peaks are almost equal in intensity (Figure 1, upper panels). Notably, these soluble purple/blue molecules (Figure S2) were obtained directly via DA and LD dissolution in a proper solvent system without enzymatic and chemical oxidation, transition metals, or the acetylation of 5,6-dihydroxyindoles, which was previously required to achieve soluble fractions of melanochrome for the analyses [12,13,14]. Differently, there was very low absorbance for NE in the spectral region associated with melanochrome formation, showing instead a larger absorbance band with a less defined λ_max_ value of approximately 340–360 nm (Figure 1). This broad band covers the visible region, conferring a yellow color to the solutions. A similar peak at 340–360 nm also appeared after the oxidation of DA and LD, reaching roughly 50% or 100% of the corresponding melanochrome absorbance, respectively. According to literature data and the results from mass spectrometry (see above) the absorbance band with a λ_max_ about 340–360 nm was ascribed to IQ, here appearing stable, differently from any previous characterization attempt confined to 5,6-dihydroxyindole analogues presenting substituents at the reactive positions of the indole ring [9,15,16,17,18,19]. Finally, the melanochrome formation appears faster for LD than for DA (Figure 1, lower panels), reaching a plateau after 1 h of reaction at 25 °C for LD only.

Considering the equal boundary conditions here explored for synthesis and analysis, in terms of concentrations, temperature, solvents, ionic strength, and pH, we ascribed the differences described above between spectra to the different abundance of reaction products, depending on the substituents of the catecholamine monomer, i.e., the α-carboxylic group for LD, and the β-hydroxyl group for NE. These findings for DA and LD oxidation, in fact, agree with previous studies carried out on DHI and DHICA, where the oxidative coupling led to several melanochrome isomers, with the same m/z, with different kinetics of formation [18,23,24,25]. Analogously, our results on the oxidation of NE agree with the evidence found in the literature that this neurotransmitter, although able to generate synthetic or natural melanins, evades the melanochrome formation reported for DA through a different oxidative pathway [26,27].

Taken together, the results reported in this paper could provide further insights into the biological route to melanins. In fact, the large amount of IQ here detected for NE oxidation underlined that IQ formation is not sufficient for the melanochrome synthesis. In particular, considering that the formation of the 2,2′-, 2,4′-, and 2.7′-biindolyls, i.e., the melanochrome isomers, previously isolated through the oxidation of DHI [12,13,14,23], was associated with the nucleophilic attack of DHI molecule to the electrophile molecule IQ, it appears, at least in our conditions, that the redox equilibrium of indole derivatives upon NE oxidation is largely dominated by IQ, largely limiting the nucleophilic reagent concentration and thus impairing the melanochrome formation.

### 3.3. Quantification of Catecholamines in Urine

**Figure 2:**
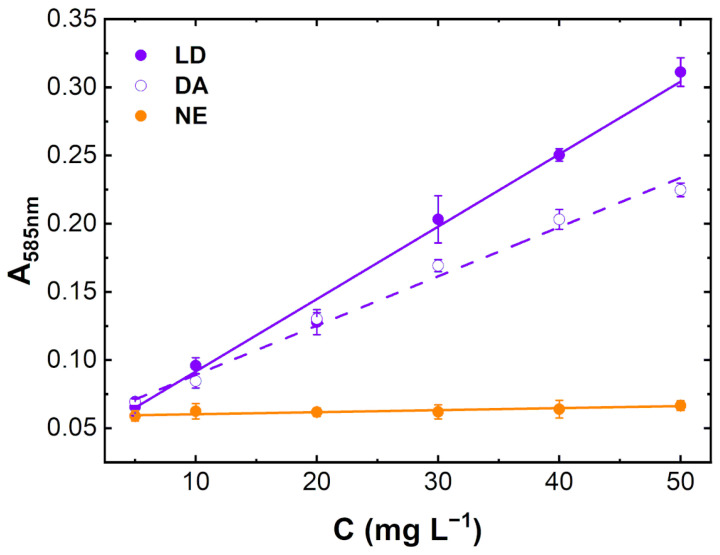
Catecholamines calibration curves in human urine spiked with levodopa (LD, purple circles), dopamine (DA, white circles), and norepinephrine (NE, orange circles), from 5.0 to 50.0 mg L^−1^, and dilute 1:8 (*v*/*v*) with a proper buffer (150 mM Mg(Ac)_2_, 150 mM NH_4_Cl at pH 9.4 in DMSO:H_2_O 1:1 (*v*/*v*)) [4]. Absorbance at 585 nm was acquired at 25 °C after 15 min of solution mixing. Each point represents the mean ± SD (*n* = 4).

The melanochrome was used as a selective colorimetric reporter, developing a quantitative diagnostic assay for DA and LD in urine. The experimental procedure was firstly optimized for levodopa in artificial urine (AU), obtaining the maximum absorbance signal at 585 nm, i.e., the best available optical filter for melanochrome detection, at an AU-to-buffer volume ratio of 1:8. The same experimental conditions were applied to the melanochrome generation in human urine spiked with DA, LD, and NE up to 50.0 mg L^−1^, covering the concentration range of DA and LD found in urine samples from Parkinson’s patients undergoing LD-based pharmacological therapy [21,22]. After 15 min, NE did not generate the purple color at any catecholamine monomer concentration, spanning from 5.0 to 50.0 mg L^−1^, as expected from zero absorbance after 15 min at 585 nm for NE in Figure 1. Conversely, the real human urine matrix did not impair the melanochrome formation in the presence of DA and LD, as showed by the linear concentration-dependent signal at 585 nm (Figure 2), where the absorbance signals are close to the values obtained in buffer after 15 min (Figure 1), demonstrating a limited matrix effect on the selective color development. The absorbance values for LD oxidation appear larger than those for DA at this reaction time, which is in further agreement with the kinetics of melanochrome formation showed in Figure 1 (lower panels). The linear fitting of calibration curves in Figure 2 gave good results (Table S1), with limits of detection (LOD) of 3.69 ± 0.17 mg L^−1^ and 2.51 ± 0.08 mg L^−1^ for DA and LD, respectively, which are lower than the minimum catecholamine concentration found in the literature for urine upon pharmacological therapy (27.75–56.57 mg L^−1^ for DA and 3.73–33.48 mg L^−1^ for LD [22]), being close to free dopamine levels found in healthy volunteers (1.71–3.22 mg L^−1^ [22]), previously reported to be, on average, 10 times lower than those found in urine samples from patients treated with levodopa [21,22]. The reproducibility of data in Figure 2, expressed as RSD_av_%, was 3.7% and 6.1% for DA and LD, respectively (Table S1). These excellent analytical performances could pave the way for the facile quantification of DA and LD in clinically relevant diagnostics and in therapeutic drug monitoring (TDM).

## 4. Conclusions

We demonstrated the selective direct formation of soluble melanochrome from dopamine and levodopa, compatible with the formation of purple/blue biindolyl molecules, previously indicated for the elusive enzymatic product of oxidation of these neurotransmitters. Moreover, we found that norepinephrine in the same condition gave only the 5,6-indolequinone monomer, previously characterized only for analogues model molecules. We thus determined the molecular weight of MC and IQ and showed their formation kinetics. This controlled generation of melanochrome was further quantified in spiked human urine samples within the range of DA and LD found in urine post-LD-based drugs, achieving very good analytical performances (RSD_av_% 3.7% and LOD 3.69 ± 0.17 mg L^−1^ for DA, RSD_av_% 6.1% and LOD 2.51 ± 0.08 mg L^−1^ for LD), and thus appearing to be very useful for the sensitive, selective, reproducible, low-cost, and non-invasive monitoring of therapy of Parkinson’s disease; the therapeutic drug monitoring allows the testing of inappropriate dosage that limits the benefits or generates toxic effects [28]. Recently, it has been reported that the chemical modification of phenolic and primary amine functional groups enabled the mapping of the dopamine and serotonin pathways, together with other neurotransmitters, metabolites, and amino acids via MALDI–MS imaging [29]. In this framework, the direct and selective melanochrome formation in situ from dopamine, as reported in the present study, may also contribute to better characterize the dopamine pathway using the highly sensitive mass spectrometry imaging applied to ex-vivo tissue specimen from the human brain with positive impacts towards more effective attempts in the pharmacological treatment of motor symptoms of Parkinson’s disease [29,30].

## Figures and Tables

**Table 1:** Results of LC-MS/MS analysis with MRM parameters in positive-ion mode.

Molecule	Precursor Ion [M+H]^+^	Product Ions	Collision Energy (eV)
DA	154.1	137.2	10
		91.1	25
		65.2	35
LD	198.1	152.1	10
		139.0	15
		135.0	15
		107.0	35
		79.0	35
^1^ MC	293.1	214.9	15
		136.9	15
		121.9	25
		58.9	30
NE	170.1	107.1	15
		135.0	15
		151.8	15
^2^ IQ	147.2	103.2	10
		73.9	20

^1^ Melanochrome from DA or LD. ^2^ 5,6-indolequinone from NE.

## Data Availability

Data available on request.

## Supplementary Materials

The following supporting information can be downloaded at: https://www.mdpi.com/article/10.3390/s23083971/s1, Figure S1: MRM chromatograms; Figure S2: Time-dependent oxidation of catecholamines at 25 °C (Spectra in Figure 1). Chosen pictures of dopamine (DA1 and DA2), norepinephrine (NE1 and NE2), and levodopa (LD1 and LD2) 0.2 g L^−1^ in (1) DMSO:H_2_O 1:1 (*v*/*v*) or (2) H_2_O in presence of 150 mM Mg(Ac)_2_, 150 mM NH_4_Cl at pH 9.4 after 2 min,10 min, 30 min, and 60 min; Table S1: Dopamine and levodopa colorimetric quantification in human urine (Figure 2).
