# Supplementary material for: Sensing of Catecholamine in Human Urine Using a Simple Colorimetric Assay Based on Direct Melanochrome and Indolequinone Formation
Source: Sensors (Basel). 2023 Apr 13;23(8):3971. doi: 10.3390/s23083971 (PMC10146333; doi:10.3390/s23083971)
Supplement: Supplementary file 1 [file sensors-23-03971-s001.zip › sensors-2286491-supplementary.pdf]

Supplementary Materials

Sensing of Catecholamine in Human Urine Using a Simple Colorimetric Assay Based on Direct Melanochrome and Indolequinone Formation

Mariagrazia Lettieri [1], Michele Spinelli [2], Laura Caponi [3], Simona Scarano [1], Pasquale Palladino [1,*], Angela Amoresano [2,4,*] and Maria Minunni [1]

[1] Department of Chemistry 'Ugo Schiff', University of Florence, 50019 Sesto Fiorentino, Italy
[2] Department of Chemical Sciences, University of Naples Federico II, 80126 Naples, Italy
[3] Laboratory of Clinical Pathology, University Hospital of Pisa, 56126 Pisa, Italy
[4] INBB, Istituto Nazionale Biostrutture e Biosistemi, Consorzio Interuniversitario, 00136 Rome, Italy
* Correspondence: pasquale.palladino@unifi.it (P.P.); angela.amoresano@unina.it (A.A.)

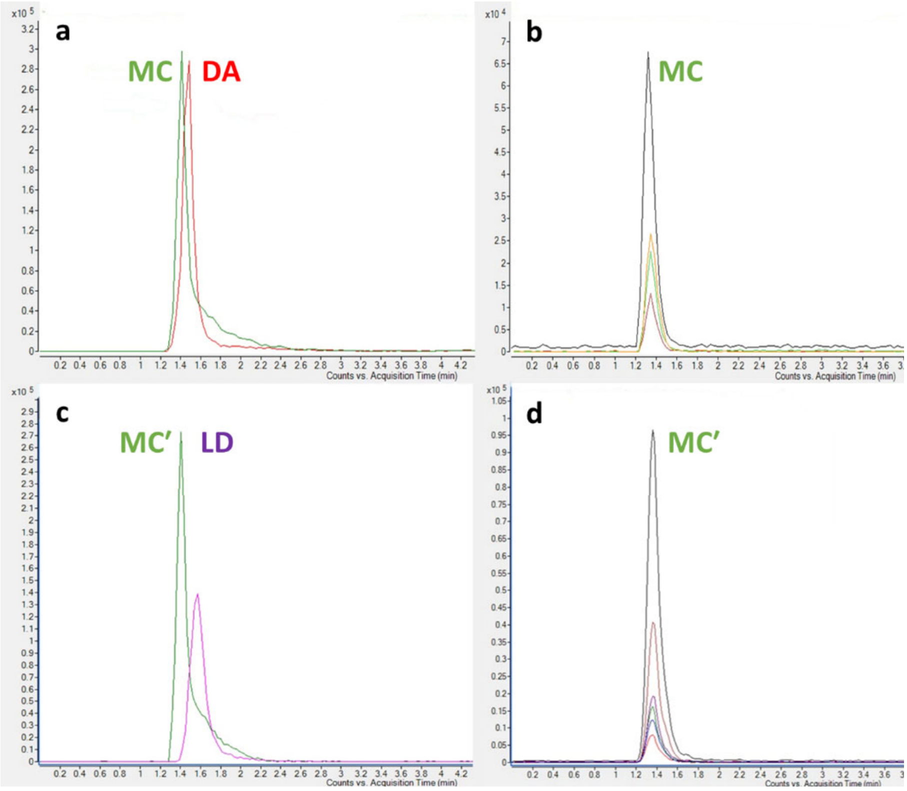

Figure S1. MRM chromatograms. (a) Catecholamine standard DA superimposed to the melanochrome molecules MC from DA. (b) MC transitions. (c) Catecholamine standard LD superimposed to the melanochrome molecules MC' from LD. (d) MC' transitions.

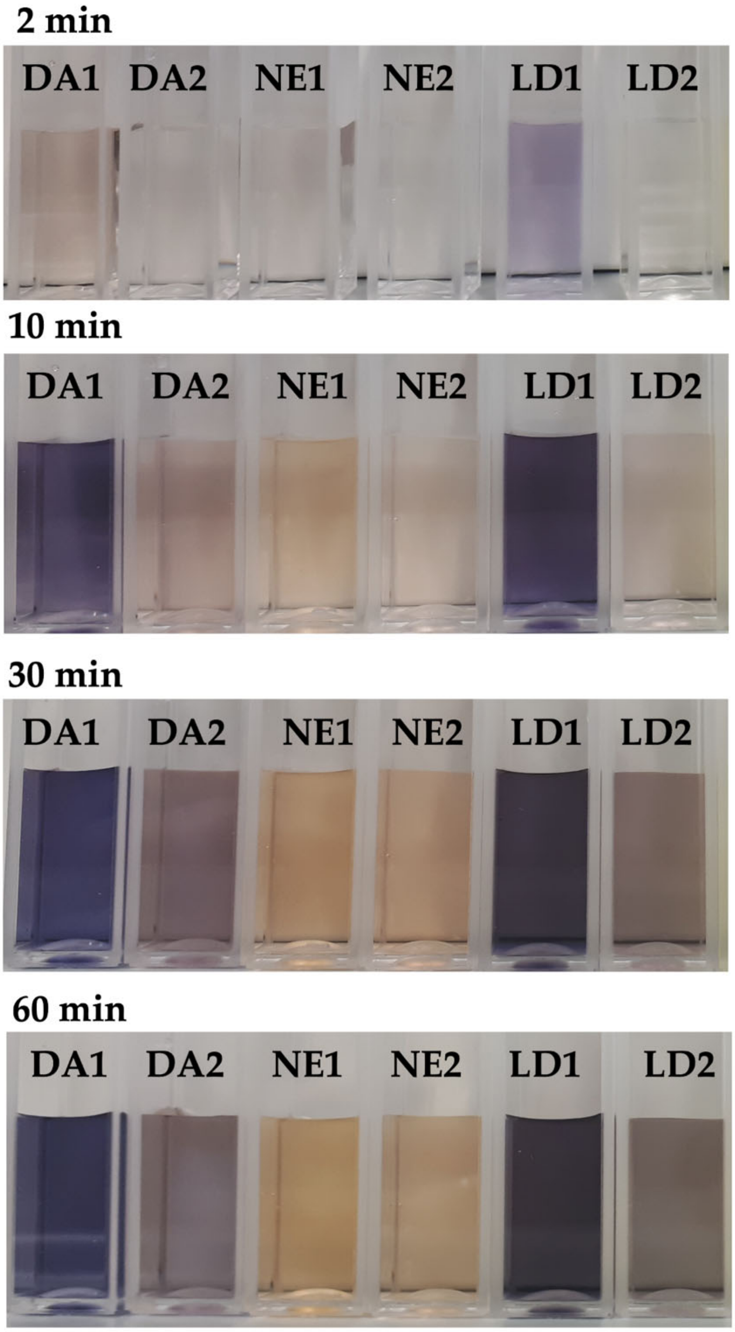

Figure S2. Time-dependent oxidation of catecholamines at 25 °C (Spectra in Figure 1). Chosen pictures of dopamine (DA1 and DA2), norepinephrine (NE1 and NE2), and levodopa (LD1 and LD2) 0.2 g L^{-1} in (1) DMSO:H_2O 1:1 (v/v) or (2) H_2O in presence of 150 mM $Mg(Ac)_2$, 150 mM NH_4Cl at pH 9.4 after 2 min,10 min, 30 min, and 60 min.

Table S1. Dopamine and levodopa colorimetric quantification in human urine (Figure 2).

Catecholamine	[1]m (L g^{-1})	a	LOD (mg L^{-1})	LOQ (mg L^{-1})	R^2	[2]RSD_{av}(%)
**DA**	3.61 ± 0.17	0.053 ± 0.004	3.69 ± 0.17	12.3 ± 0.6	0.991	3.7
**LD**	5.32 ± 0.18	0.038 ± 0.005	2.51 ± 0.09	8.4 ± 0.3	0.994	6.1

[1] Fitting equation 1: $A_{585nm} = m \times C + a$

[2] Calculated between 5.0 mg L^{-1} and 50.0 mg L^{-1} of DA or LD spiked in human urine.

For abbreviations meaning, refer to Materials and Methods.
